# Mathematical modeling of Myosin induced bistability of Lamellipodial fragments

**DOI:** 10.1007/s00285-016-1008-2

**Published:** 2016-04-25

**Authors:** S. Hirsch, A. Manhart, C. Schmeiser

**Affiliations:** 1 Faculty of Mathematics, University of Vienna, Oskar-Morgenstern-Platz 1, 1090 Vienna, Austria; 2 Courant Institute of Mathematical Sciences, New York University, 251 Mercer Street, 10012 New York, USA

**Keywords:** Actin, Mathematical model, Cytoskeleton, 35Q92, 34D20, 92C17, 00A72

## Abstract

For various cell types and for lamellipodial fragments on flat surfaces, externally induced and spontaneous transitions between symmetric nonmoving states and polarized migration have been observed. This behavior is indicative of bistability of the cytoskeleton dynamics. In this work, the Filament Based Lamellipodium Model (FBLM), a two-dimensional, anisotropic, two-phase continuum model for the dynamics of the actin filament network in lamellipodia, is extended by a new description of actin–myosin interaction. For appropriately chosen parameter values, the resulting model has bistable dynamics with stable states showing the qualitative features observed in experiments. This is demonstrated by numerical simulations and by an analysis of a strongly simplified version of the FBLM with rigid filaments and planar lamellipodia at the cell front and rear.

**Electronic supplementary material:**

The online version of this article (doi:10.1007/s00285-016-1008-2) contains supplementary material, which is available to authorized users.

## Introduction

In a variety of physiological processes such as wound healing, immune response, or embryonic development, crawling cells play a vital role (Ananthakrishnan and Ehrlicher 2007). Cell motility is the result of an interplay between protrusion at the ’front’ edge of the cell (w.r.t. the direction of movement), retraction at the rear, as well as translocation of the cell body (Small and Resch 2005). It only occurs when the cell is polarized with a front and a differently shaped rear (Kozlov and Mogilner 2007).

Both protrusion and retraction involve the so-called *lamellipodium*, a thin, sheet-like structure along the perimeter of a cell, consisting of a meshwork of *actin filaments*. F-actin is a polar dimer that forms inextensible filaments with a fast-growing plus (barbed) end and a slow-growing minus (pointed) end (Holmes et al. 1990).

The barbed ends abut on the membrane at the leading edge (Mogilner 2009) and have a high probability of polymerization (i.e. elongation of the filament by insertion of new actin monomers), whereas at the pointed ends mostly depolymerization (removal of one monomer) or disassembly of larger parts through severing of the filament occurs. Once a balance between polymerization and depolymerization is reached, each incorporated monomer is being pushed back by newly added monomers. Using the filament itself as a frame of reference, this can be described as movement of monomers from the barbed end towards the pointed end, a process called *treadmilling* (see Manhart et al. 2015a and the references therein for an overview of the involved processes and proteins). New filaments are nucleated predominantely by branching off existing filaments. The resulting meshwork is an (almost) two-dimensional array of (almost) diagonally arranged actin filaments with decreasing density towards the cell body (Small et al. 1995; Vinzenz 2012).

The lamellipodium is stabilized by the cell membrane (surrounding the entire cell Mitchison and Cramer 1996; Vallotton et al. 2005), adhesions to the substrate (Li et al. 2003; Pierini et al. 2000), cross-linking proteins (Nakamura et al. 2007; Schwaiger et al. 2004) and myosin II filaments (Svitkina et al. 1997), the latter two binding to pairs of filaments. Some of the long filaments from the lamellipodium extend into the region behind, where (through the contractile effect of myosin II) forces are generated which pull the lamellipodium backwards (Small and Resch 2005).

Fish epidermal keratocytes are fast-moving cells with a relatively simple shape (circular, when stationary and crescent-moon-shaped, when moving Lee et al. 1993), which makes them ideal subjects for analysis. Furthermore, they exhibit a lamellipodium with a smooth edge and a fairly uniform distribution of filaments (Lacayo et al. 2007; Small and Resch 2005; Theriot and Mitchison 1991). During the transition from the stationary to the moving state, the lamellipodium in the rear of the cell collapses and the *rear bundle* is formed, where myosin II generates a contractile force (Svitkina et al. 1997; Tojkander et al. 2012; Verkhovsky et al. 1997).

**Fig. 1:**
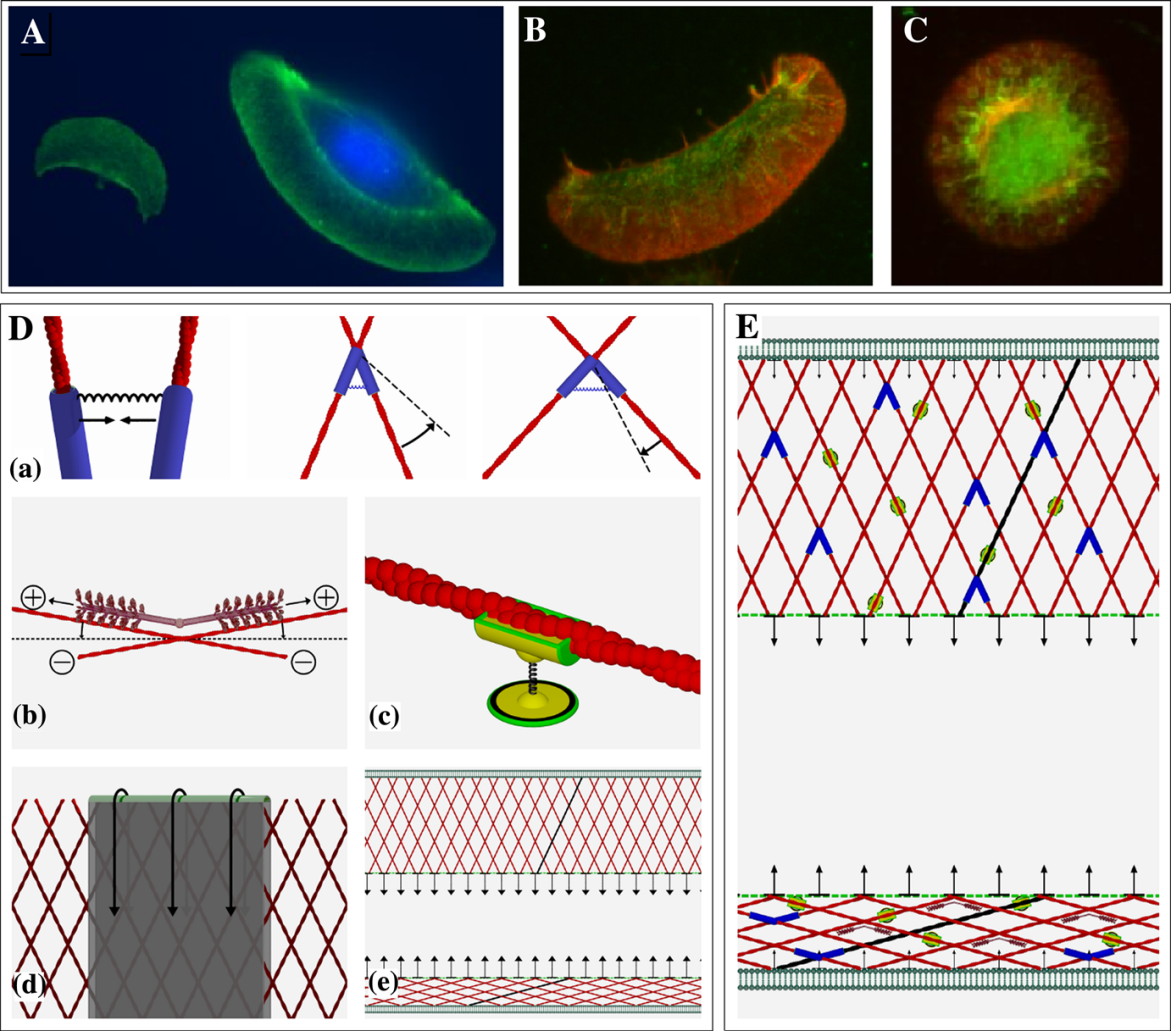
**a** A moving keratocyte (*right*) and a moving cytoplast (*left*), actin is labelled in *green*, the nucleus in *blue*. **b, c** A moving and a stationary cytoplast (fragment), respectively. The actin network is labelled in *red*, myosin in *green*. **a, b, c** Are reproduced from (Manhart 2011). **e** Idealization with protruding lamellipodium at the top and lamellipodium collapsed by actin–myosin interaction at the bottom. **d** Model ingredients of the simplified FBLM (clockwise, starting *top left*) cross-link stretching, cross-link twisting, filament-substrate adhesion, connection between front and rear by stress fibres, membrane stretching, actin–myosin interaction (color figure online)

Treatment with staurosporine (a protein kinase inhibitor) results in the formation of completely detached lamellipodial fragments, lacking a cell body, microtubules and most other cell organelles. Remarkably, these fragments can either remain stationary while adopting a circular shape, or can move on their own, adapting their appearance to the same crescent-moon shape as the keratocyte itself (Kozlov and Mogilner 2007; Verkhovsky et al. 1999) (see Fig. 1a–c). This suggests that the necessary ingredients for movement are all present in the lamellipodium (until it runs out of energy).

Various approaches to continuum mechanical modeling of the lamellipodium exist (see e.g. Kruse et al. 2006; Rubinstein et al. 2005). Bistability results similar to this work have been obtained in Ziebert et al. (2012), where a phase-field approach is used to describe the interplay between the cell shape, the mean orientation of the filaments within the network and the actin–myosin interaction. A strongly simplified model is developed in Kozlov and Mogilner (2007), where bistability has been obtained analytically as the consequence of the properties of a free energy functional containing contributions from the lamellipodium and a possible rear bundle.

This work is based on the FBLM (Manhart et al. 2015a; Ölz and Schmeiser 2010a, b), a two-dimensional, anisotropic, two-phase model derived from a microscopic (i.e. individual filament based) description, accounting for most of the phenomena mentioned above. It describes the actin network in terms of two transversal families of locally parallel filaments, stabilized by transient cross-links and substrate adhesions. In Sect. 2 the FBLM is presented and extended by a model for actin–myosin interaction between the two families. We assume that myosin filaments can connect only when the families are anti-parallel enough and they are described as transient, similar to cross-links. They tend to slide the two families relative to each other, and they are assumed to have a turning effect, making the two families more anti-parallel. The derivation of the additional myosin terms is presented in detail. Readers not consulting (Manhart et al. 2015a; Ölz and Schmeiser 2010a, b) may take this as representative also for the derivation of the adhesion and cross-link models.

The properties of the actin–myosin model are expected to produce the desired bistable behavior. This is demonstrated by numerical simulations in Sect. 8, which indicate the existence of two stable states, a rotationally symmetric nonmoving state and a polarized state, where the cell moves. The moving state is characterized by a more anti-parallel network in the rear of the cell, where actin–myosin interaction is active. Complete collapse of the network and consequential generation of a rear bundle are avoided, since the FBLM is (so far) unable to deal with such topological changes.

The occurrence of bistability is also proven analytically for a strongly simplified model. In Sect. 3 the complexity of the model is reduced in a first step by assuming rigid filaments. Then a planar, translationally invariant lamellipodium is considered in Sect. 4, which reduces the model to a system of three ordinary differential equations. Here we also neglect the effects of branching and capping, assumed to be in equilibrium, as well as filament severing within the modelled part of the lamellipodium, implying a constant actin density there. Bistability is obtained for this model in Sect. 5. Finally, in Sect. 6 a cell (fragment) is replaced by a pair of connected back-to-back planar lamellipodia, and the existence of stable stationary (symmetric) as well as moving (polarized) states is proven. The same bistable behavior is observed in the simulations of the full model in Sect. 8.

Figure 1 depicts the main components of the simplified version of the FBLM (D and E) together with one keratocyte and three fragments (A–C). The crescent-moon shaped cells and cell fragments are moving, whereas the circularly shaped fragment remains stationary. One can also observe that in moving fragments myosin can predominantely be found at the cell rear. In Fig. 1e, the idealized model obtained in Sects. 3, 4, 5, 6 is illustrated. It can be interpreted as description of lamellipodial sections at the front and at the rear of the cell. The main model ingredients are depicted in Fig. 1d: (e) diagonally arranged filaments (red) together with the membrane (dark green) and arrows indicating inward pulling forces due to stress fibers in the interior of the cell (dashed green line), (d) the cell membrane (green, with arrows indicating the force acting on the barbed ends due to membrane tension), (a) cross-links [blue, producing friction between the filament families and a turning force trying to establish an equilibrium angle—arrows show the forces acting on the cross links due to resistance to stretching and bending above or below a certain angle (visualized by the black dashed line)], (b) myosin filaments (pink, trying to slide the filament families and to make them anti-parallel—straight arrows indicate that myosin moves towards the plus ends along both filaments while curved arrows illustrate the tendency to establish an angle of $180^\circ$ between the filaments, analogously to the cross-links), (c) adhesions (yellow, connecting a filament through the membrane with the substrate thus producing friction relative to the substrate).

## Adding actin–myosin interaction to the Filament Based Lamellipodium Model (FBLM)

Our starting point is the FBLM as introduced in Ölz and Schmeiser (2010a) (see also Manhart et al. 2015a):

1 $$\begin{aligned} 0= & {} \mu ^B \partial _s^2\left( \eta \partial _s^2 F\right) + \mu ^A \eta D_t F - \partial _s\left( \eta \lambda _\text {inext} \partial _s F\right) \nonumber \\&+\,\widehat{\mu ^S}\eta \eta ^* (D_t F - D_t^* F^*) \pm \partial _s\left( \widehat{\mu ^T}\eta \eta ^* (\varphi -\varphi _0)\partial _s F^{\perp }\right) , \end{aligned}$$

where $F=F(\alpha ,s,t)\in \mathbb {R}^2$ describes the position and deformation of actin filaments in the plane at time *t*. More precisely, the variable $\alpha \in A\subset \mathbb {R}$, for some interval *A*, is a filament label, and $s\in [-L(\alpha ,t),0]$ denotes an arclength parameter along filaments, which means that the constraint

2 $$\begin{aligned} |\partial _s F| = 1 \end{aligned}$$

has to be satisfied. Here $L(\alpha ,t)$ is the maximal length of filaments in an infinitesimal region $d\alpha$ around $\alpha$. The filament length density with respect to $\alpha$ and *s* is given by $\eta (\alpha ,s,t)$, which will be assumed as given (see Manhart et al. 2015a for a dynamic model incorporating polymerization, depolymerization, nucleation, and branching effects). The value $s=0$ corresponds to the so called *barbed ends* of the polar filaments, abutting the leading edge of the lamellipodium. The rear boundary $s=-L(\alpha ,t)$ is introduced somewhat artificially since the rear end of the lamellipodium is typically not well defined. By polymerization with speed $v(\alpha ,t)$ (also assumed as given in this work), monomers move along filaments in the negative *s*-direction. Their speed relative to the nonmoving substrate is therefore given by $D_t F$ with the material derivative $D_t = \partial _t - v\partial _s$.

The terms in the first line of (1) correspond to the filaments’ resistance against bending with stiffness parameter $\mu ^B$, to friction relative to the substrate as a consequence of adhesion dynamics with adhesion coefficient $\mu ^A$, and to the constraint (2) with the Lagrange multiplier $\lambda _\text {inext}$.

The FBLM is actually a two phase model, and *F* may stand for either of the two families $F^+$ or $F^-$. The terms in the second line of (1) describe the interaction between the two families, with the other family indicated by the superscript $*$. The interaction is the consequence of dynamic cross-linking and leads to a friction term proportional to the relative velocity between the two families and to a turning force trying to push the angle $\varphi$ ($\cos \varphi = \partial _s F\cdot \partial _s F^*$) between crossing filaments to its equilibrium value $\varphi _0$, corresponding to the equilibrium conformation of the cross-linker molecule ($F^\bot = (-F_y,F_x)$). The $*$-quantities corresponding to the other family have to be evaluated at $(\alpha ^*,s^*)$, determined by the requirement $F(\alpha ,s,t)= F^*(\alpha ^*,s^*,t)$. It is a basic geometric modeling assumption that the coordinate change $(\alpha ,s) \leftrightarrow (\alpha ^*,s^*)$ is one-to-one, wherever the two families overlap. It requires that filaments of the same family do not cross each other and that pairs of filaments of different families cross each other at most once. Finally, the coefficients are given by

3 $$\begin{aligned} \widehat{\mu ^S} = \mu ^S \left| \frac{\partial {\alpha }^*}{\partial s}\right| ,\quad \widehat{\mu ^T} = \mu ^T \left| \frac{\partial {\alpha }^*}{\partial s}\right| , \end{aligned}$$

with constants $\mu ^{S,T}$, wherever *F* crosses another filament, and zero elsewhere. The partial derivative refers to the coordinate transformation introduced above.

The FBLM will be extended by the effects of myosin polymers. The basic assumption is that pairs of crossing actin filaments, which lie antiparallel enough, may be connected by a bipolar myosin filament. The modeling is similar to that of cross-links and we will give details of the derivation of the myosin related term (originally presented in Manhart 2011) as an example of the general modeling strategy of the FBLM. We assume the following about myosin molecules:

- Myosin filaments connect pairs of crossing actin filaments.
- Due to the motor activity of the myosin heads they “walk” towards the barbed ends of actin filaments with a fixed speed $v^M$.
- The rates of creation and breakage of the myosin connections depend on the forces acting on them (see below).
- The connections exert twisting forces on the connected actin filaments towards the equilibrium angle $\pi$, i.e. the antiparallel state. These forces are caused by the stiffness of myosin filaments.
- The connections can be stretched against an elastic restoring force.

The key to the bistability results presented in the next sections is the assumption that the myosin activity (specified below) depends on the angle between crossing actin filaments. This is supported by data reported in Reymann (2012), where it has been shown that myosin can act much more efficiently on antiparallel actin networks as opposed to branched networks. In the original model of the myosin dynamics presented in Manhart (2011), the growth of myosin filaments was also included. Here, we omit myosin size effects for the sake of simplicity. The derivation is done in five steps.

*Step 1: Energy contributions* Let a filament with label $\alpha$ have a crossing with a filament from the other family with label $\alpha ^*$. By $s(\alpha ,\alpha ^*,t)$ we denote the *s*-position along the $\alpha$-filament where this crossing occurs. If at time $t-a$ a myosin filament has been attached at this crossing (i.e. the connection has age *a* at time *t*), the *s*-value of the binding site will change due to the aforementioned treadmilling effect (caused by actin polymerization) and the myosin motor activity. Therefore at time *t* the myosin filament binds to the actin filament at

4 $$\begin{aligned} s_a(\alpha , \alpha ^*,t)=s(\alpha , \alpha ^*,t-a)-\int _{t-a}^t\left( v(\alpha ,\tilde{t})-v^M\right) d\tilde{t}. \end{aligned}$$

Analogously, the position $s_a^*(\alpha , \alpha ^*,t)$ at time *t* of the binding site on the $\alpha ^*$-filament is computed. With the aid of $s_a$ and $s_a^*$ we can now determine the deviations from the unstretched and untwisted equilibrium state of the myosin filament:

$$\begin{aligned} S_a^M(\alpha , \alpha ^*, t)= & {} F\left( \alpha , s_a(\alpha , \alpha ^*,t),t\right) -F^*\left( \alpha ^*, s_a^*(\alpha , \alpha ^*,t),t\right) ,\\ T_a^M(\alpha , \alpha ^*, t)= & {} \varphi _a(\alpha , \alpha ^*, t)-\pi ,\quad \text {where}\\ \cos (\varphi _a(\alpha , \alpha ^*, t))= & {} \partial _s F\left( \alpha , s_a(\alpha , \alpha ^*,t),t\right) \cdot \partial _s F^*\left( \alpha ^*, s_a^*(\alpha , \alpha ^*,t),t\right) . \end{aligned}$$

Note that $S_0^M=0$ and $T_0^M=\varphi -\pi$, i.e. the myosin filament is unstretchted but possibly bent at the time a connection is created.

Now let $\rho ^M(\alpha , \alpha ^*, a, t)$ denote the probability density with respect to age *a* of an active myosin connection between the filaments with label $\alpha$ and $\alpha ^*$ at time *t*. Following classical modeling for age-structured populations we assume $\rho ^M$ to satisfy

$$\begin{aligned} \partial _t \rho ^M+\partial _a \rho ^M= & {} -\zeta (S_a^M, T_a^M)\rho ^M,\\ \rho ^M(a=0)= & {} \beta (T_0^M)\left( 1-\int _0^\infty \rho ^M d \tilde{a} \right) . \end{aligned}$$

The rate of breakage $\zeta$ may depend on the stretching and twisting forces acting on the myosin filament. We also allow for the possibility that the rate of creation $\beta$ is affected by how favorable the angle between the actin filaments is. The interpretation of the last factor is that a new connection can only connect a so far unconnected pair of actin filaments.

The main Eq. (1) of the FBLM is the result of a variational approach applied to the problem of minimizing a sum of potential energies. Each energy contribution produces forces acting on the actin filament (i.e. bending stiffness, adhesions, etc.). To include new terms, it is therefore necessary to formulate the corresponding energy. For the elastic stretching and twisting of the myosin filaments, we choose the form

$$\begin{aligned} U^M_\text {stretching}\left[ F, F^*\right]&=\int _0^\infty \int _{\mathcal {C}(t-a)}\frac{\kappa ^{SM}}{2}\left| S_a^M\right| ^2\rho ^M\eta \eta ^* d(\alpha , \alpha ^*) da,\\ U^M_\text {twisting}\left[ F, F^*\right]&=\int _0^\infty \int _{\mathcal {C}(t-a)}\frac{\kappa ^{TM}}{2}(T_a^M)^2\rho ^M\eta \eta ^* d(\alpha , \alpha ^*) da. \end{aligned}$$

The integration domain $\mathcal {C}(t-a)$ includes all pairs of filaments $(\alpha , \alpha ^*)$, which had a crossing at time $t-a$. The constants $\kappa ^{SM}$ and $\kappa ^{TM}$ are elasticity coefficients.

*Step 2: Scaling* The key scaling assumption of the original FBLM is that the average lifetime of a cross-link or adhesion is small compared to the average time a monomer spends inside a filament, a ratio denoted by $\varepsilon$. In the limit $\varepsilon \rightarrow 0$ the non-locality in time is removed from the problem. Furthermore this reduces the effect of adhesions to friction with the substrate and that of the cross-linkers to friction between the filament families (compare Eq. (1)).

This raises the question of what to assume about the average lifetime of a myosin filament. In Svitkina et al. (1997) it was observed that in stationary cytoplasts myosin spots stay small and disappear after some time, indicating their transient nature. The situation is quite different for moving cytoplasts, where the myosin spots grow in size over time. Since the aim of this paper is to describe the onset of movement, we use here the same scaling assumption as for cross-links, i.e. that the lifetime of a myosin filament is relatively short. Without going into further detail about the precise reference values for all variables and parameters, we state the resulting energy contributions and equations after scaling: The density equations take the form

$$\begin{aligned} \varepsilon \partial _t \rho _\varepsilon ^M+\partial _a \rho _\varepsilon ^M= & {} -\zeta (S_{\varepsilon a}^M, T_{\varepsilon a}^M)\rho _\varepsilon ^M,\\ \rho _\varepsilon ^M(a=0)= & {} \beta (T_0^M)\left( 1-\int _0^\infty \rho _\varepsilon ^M d \tilde{a} \right) . \end{aligned}$$

Since $S_{\varepsilon a}^M\rightarrow 0$ and $T_{\varepsilon a}^M\rightarrow T_0^M=\varphi -\pi$ as $\varepsilon \rightarrow 0$, we can explicitly calculate the solution to the limiting equation. In the following we will replace dependencies on $T_0^M$ by dependencies on $\varphi$. Ignoring a possible initial time layer, the limiting solution is given by

5 $$\begin{aligned} \rho _0^M=\frac{\beta (\varphi ) \zeta (0,\varphi )}{\beta (\varphi )+\zeta (0, \varphi )}e^{-\zeta (0,\varphi )a}. \end{aligned}$$

Note that $\rho _0^M$ depends on *t* and the indices $\alpha$ and $\alpha ^*$ via the angle between the filaments.

The scaled energy contributions take the form

$$\begin{aligned} U^M_\text {stretching}\left[ F, F^*\right]&=\frac{1}{\varepsilon }\int _0^\infty \int _{\mathcal {C}(t-\varepsilon a)}\frac{\kappa ^{SM}}{2}\left| S_{a\varepsilon }^M\right| ^2\rho _\varepsilon ^M\eta \eta ^* d(\alpha , \alpha ^*) da,\\ U^M_\text {twisting}\left[ F, F^*\right]&=\int _0^\infty \int _{\mathcal {C}(t-\varepsilon a)}\frac{\kappa ^{TM}}{2}(T_{\varepsilon a}^M)^2\rho _\varepsilon ^M\eta \eta ^* d(\alpha , \alpha ^*) da. \end{aligned}$$

The factor $1/\varepsilon$ in front of the stretching contribution is the result of a scaling assumption. It ensures that the effect of myosin filament stretching does not vanish in the limit, although the energy contribution itself does.

*Step 3: Variation and macroscopic limit* The next step in the derivation is to calculate the variations $\delta U[F, F^*]\delta F$ of the energy contributions, considering $\rho _\varepsilon ^M$ given at this stage. After passing to the limit $\varepsilon \rightarrow 0$, we obtain

$$\begin{aligned} \delta U^M_\text {stretching}\left[ F, F^*\right] \delta F&=\int _{\mathcal {C}(t)}\mu ^{SM}\left( D_t^MF-D_t^{M*} F^*\right) \cdot \delta F \,\eta \eta ^* d(\alpha , \alpha ^*),\\ \delta U^M_\text {twisting}\left[ F, F^*\right] \delta F&=\int _{\mathcal {C}(t)}\mu ^{TM}\left( \partial _s F^\perp \cdot \partial _s \delta F\right) (\varphi -\pi ) \eta \eta ^* d(\alpha , \alpha ^*), \end{aligned}$$

where $D_t^M F$ denotes the velocity of a myosin binding site on the actin filament relative to the substrate, with

$$\begin{aligned} D_t^M:=\partial _t-(v-v^M)\partial _s. \end{aligned}$$

The stiffness parameters $\mu ^{SM}$ and $\mu ^{TM}$ result from using the representation of $\rho _0^M$ given in (5) to evaluate the integrals with respect to the myosin age *a*. They take the form

$$\begin{aligned} \mu ^{SM}(\varphi )=\frac{\beta (\varphi )\kappa ^{SM}}{ \zeta (0,\varphi )\left( \beta (\varphi )+\zeta (0,\varphi )\right) }, \quad \mu ^{TM}(\varphi )=\frac{\beta (\varphi )\kappa ^{TM}}{\beta (\varphi )+\zeta (0, \varphi )}. \end{aligned}$$

Motivated by the findings in Reymann (2012), we assume that there exists a cutoff angle $\overline{\varphi }<\pi$ such that

$$\begin{aligned} \mu ^{SM}(\varphi ) = \mu ^{TM}(\varphi ) = 0\quad \text {for}\quad \varphi < \overline{\varphi }<\pi . \end{aligned}$$

*Step 4: Euler–Lagrange Equations* The final step in the derivation is to formulate the corresponding Euler-Lange equations. Together with the original terms given in (1), the modified model takes the form

6 $$\begin{aligned} 0= & {} \mu ^B \partial _s^2\left( \eta \partial _s^2 F\right) + \mu ^A \eta D_t F - \partial _s\left( \eta \lambda _\text {inext} \partial _s F\right) \nonumber \\&+\, \widehat{\mu ^S}\eta \eta ^* (D_t F - D_t^* F^*) \pm \partial _s\left( \widehat{\mu ^T}\eta \eta ^* (\varphi -\varphi _0)\partial _s F^{\perp }\right) \nonumber \\&+\, \widehat{\mu ^{SM}}\eta \eta ^* (D^M_t F - D^{M*}_t F^*) \pm \partial _s\left( \widehat{\mu ^{TM}}\eta \eta ^* (\varphi -\pi )\partial _s F^{\perp }\right) , \end{aligned}$$

with

7 $$\begin{aligned} \widehat{\mu ^{SM}} = \mu ^{SM}(\varphi ) \left| \frac{\partial {\alpha }^*}{\partial s}\right| ,\quad \widehat{\mu ^{TM}} = \mu ^{TM}(\varphi ) \left| \frac{\partial {\alpha }^*}{\partial s}\right| , \end{aligned}$$

The introduction of $\widehat{\mu ^{SM}}$ and $\widehat{\mu ^{TM}}$ is a consequence of the mapping between $\mathcal {C}$ and $\left\{ (\alpha ,s): \alpha \in A, s\in [-L(\alpha ,t),0]\right\}$. Further details of the model derivation can be found in Manhart (2011).

*Step 5: Boundary conditions* describe the forces acting on the filaments at their barbed ends and at the artificially introduced ends at the boundary of the modeling domain:

8 $$\begin{aligned}&\mu ^B \partial _s\left( \eta \partial _s^2 F\right) - \eta \lambda _\text {inext} \partial _s F \pm \widehat{\mu ^T}\eta \eta ^* (\varphi -\varphi _0)\partial _s F^{\perp } \pm \widehat{\mu ^{TM}}\eta \eta ^* (\varphi -\pi )\partial _s F^{\perp } \nonumber \\&\quad = -f_0, \partial _s^2 F = 0, \quad \text{ for } \quad s=0.\nonumber \\&\mu ^B \partial _s\left( \eta \partial _s^2 F\right) - \eta \lambda _\text {inext} \partial _s F \pm \widehat{\mu ^T}\eta \eta ^* (\varphi -\varphi _0)\partial _s F^{\perp } \pm \widehat{\mu ^{TM}}\eta \eta ^* (\varphi -\pi )\partial _s F^{\perp } \nonumber \\&\quad = f_L, \partial _s^2 F = 0,\quad \text{ for } \quad s=-L. \end{aligned}$$

Thus, there are no torques applied at the ends. The choice of the linear forces $f_0$ and $f_L$ along the leading edge and, respectively, along the artificial boundary will be discussed later.

## Rigid actin filaments in the limit of large bending stiffness

We want to derive a simplified model with rigid actin filaments. This is motivated on the one hand by the observation that filaments within the lamellipodium are typically rather straight (Vinzenz 2012). On the other hand stiff filaments can be interpreted as a description of only the outermost part of the lamellipodial region, where filaments are (locally) straight. The resulting model is mathematically much simpler and can be derived by assuming a relatively large bending stiffness $\mu ^B$. The limit $\mu ^B\rightarrow \infty$ will be carried out formally in this section.

The solutions of the formal limit

$$\begin{aligned} 0 = \partial _s^2\left( \eta \partial _s^2 F\right) \end{aligned}$$

of (6), together with the boundary conditions

$$\begin{aligned} \partial _s^2 F = 0, \quad \text{ for } \quad s=0,-L, \end{aligned}$$

and with the constraint (2), can be written as

9 $$\begin{aligned} F(\alpha ,s,t) = F_0(\alpha ,t) + (s-s_0(\alpha ,t))d(\omega (\alpha ,t)) ,\quad \text{ with } \quad d(\omega ) = \left( {\begin{array}{c}\cos \omega \\ \sin \omega \end{array}}\right) , \end{aligned}$$

where $s_0$ is determined by

$$\begin{aligned} \int _{-L}^0 \eta (\alpha ,s,t)(s-s_0(\alpha ,t))ds = 0. \end{aligned}$$

In other words, $F_0$ is the center of mass of the filament, and $d(\omega )$ its direction. The components of $F_0$ and the angle $\omega$ are still to be determined. The total force balance obtained by integration of (6) with respect to *s* and using the boundary conditions (8) reads

10 $$\begin{aligned} f_0 + f_L =&\int _{-L}^0 \Bigl ( \mu ^A \eta D_t F+ \widehat{\mu ^S}\eta \eta ^* (D_t F - D_t^* F^*) \nonumber \\&+ \widehat{\mu ^{SM}}\eta \eta ^* (D_t F - D_t^* F^*+ v^M(\partial _s F - \partial _s F^*))\Bigr ) ds. \end{aligned}$$

Note that it does not contain $\mu ^B$ and therefore remains valid in the limit. Similarly, the total torque balance is obtained by integration of (6) against $(F-F_0)^\bot$:

11 $$\begin{aligned}&(F-F_0)^\bot (s=0)\cdot f_0 + (F-F_0)^\bot (s=-L)\cdot f_L \nonumber \\&\quad = \mp \int _{-L}^0 \widehat{\mu ^T} \eta \eta ^*(\varphi -\varphi _0)ds \mp \int _{-L}^0 \widehat{\mu ^{TM}} \eta \eta ^*(\varphi -\pi )ds \nonumber \\&\qquad + \int _{-L}^0 (F-F_0)^\bot \cdot \Bigl ( \mu ^A \eta D_t F+ \widehat{\mu ^S}\eta \eta ^* (D_t F - D_t^* F^*) \nonumber \\&\qquad + \widehat{\mu ^{SM}}\eta \eta ^* (D_t F - D_t^* F^*+ v^M(\partial _s F - \partial _s F^*))\Bigr ) ds. \end{aligned}$$

This completes the formulation of the rigid filament version of the FBLM. Substitution of (9) into (10) and (11) gives a system of ordinary differential equations for $F_0$ and $\omega$. Note that coupling with respect to $\alpha$ happens only indirectly through the interaction between the two filament families.

## A geometric simplification: the planar lamellipodium

Since in keratocytes the leading edge is rather smooth, we approximate a piece of lamellipodium by an infinite strip, parallel to the *x*-axis, and invariant to translations and to reflection. For the given data this means that the maximal filament length *L* and the polymerization speed *v* are constants. As a further simplification, we assume no filament ends inside the modeled part of the lamellipodium with the consequence $\eta = 1$ (and $s_0=-L/2$).

We assume two families of rigid filaments (9) with

$$\begin{aligned} F_0^+(\alpha ^+,t)= & {} \left( {\begin{array}{c}x(t)+\alpha ^+\\ y(t)\end{array}}\right) ,\quad \alpha ^+\in \mathbb {R},\quad \omega ^+(\alpha ^+,t) = \omega (t) \in [0,\pi /2],\\ F_0^-(\alpha ^-,t)= & {} \left( {\begin{array}{c}-x(t)+\alpha ^-\\ y(t)\end{array}}\right) ,\quad \alpha ^-\in \mathbb {R},\quad \omega ^-(\alpha ^-,t) = \pi -\omega (t) \in [\pi /2,\pi ], \end{aligned}$$

giving

$$\begin{aligned} F^\pm (\alpha ^\pm ,s^\pm ,t) = \left( {\begin{array}{c}\pm x(t) + \alpha ^\pm \pm (s^\pm +L/2)\cos \omega (t)\\ y(t) + (s^\pm + L/2)\sin \omega (t)\end{array}}\right) \,, \alpha ^\pm \in \mathbb {R},\quad s^\pm \in [-L,0]. \end{aligned}$$

The angle between two crossing filaments and the coordinate change between the two families mentioned in Sect. 2 are easily computed:

$$\begin{aligned} \varphi =\pi -2\omega , \quad \alpha ^- = \alpha ^+ + 2x(t) + (2s^+ + L)\cos \omega (t),\quad s^- = s^+. \end{aligned}$$

It provides the geometric quantity needed in (3) and (7):

$$\begin{aligned} \left| \frac{\partial \alpha ^-}{\partial s^+}\right| = 2\cos \omega . \end{aligned}$$

This quantity can be interpreted as a measure of the density of crossings, with a maximum at $\omega =0$ (fully collapsed lamellipodium) and a minimum at $\omega =\pi /2$ (all filaments are parallel, no crossings).

With the planar lamellipodium ansatz, the Eqs. (10) and (11) become independent of $\alpha$ and constitute a system of three ordinary differential equations for the unknowns $(x(t),y(t),\omega (t))$:

12 $$\begin{aligned}&\dot{x} \left[ \mu ^A + 4(\mu ^S + \mu ^{SM}(\pi -2\omega ))\cos \omega \right] \nonumber \\&\quad = \frac{f_{0,x} + f_{L,x}}{L} + \mu ^A v \cos \omega + 4\mu ^S v \cos ^2\omega \nonumber \\&\qquad +\, 4\mu ^{SM}(\pi -2\omega )(v-v^M)\cos ^2\omega , \end{aligned}$$

13 $$\begin{aligned}&\dot{y} \mu ^A = \frac{f_{0,y} + f_{L,y}}{L} +\mu ^A v \sin \omega , \end{aligned}$$

14 $$\begin{aligned}&\dot{\omega }\left[ \mu ^A + 4\sin ^2\omega \cos \omega (\mu ^S + \mu ^{SM}(\pi -2\omega ))\right] \nonumber \\&\quad = \frac{6}{L^2} d(\omega )^\bot \cdot (f_0-f_L) + \frac{24}{L^2} \mu ^T (\pi -2\omega -\varphi _0)\cos \omega \nonumber \\&\qquad -\, \frac{48}{L^2} \mu ^{TM}(\pi -2\omega )\omega \cos \omega . \end{aligned}$$

## Forces at the filament ends-steady protrusion

The membrane stretched around the lamellipodium exerts a force on the polymerizing barbed ends. On the other hand, we assume that the filaments at the rear of the lamellipodium are connected to stress fibres pulling them backwards, another consequence of actin–myosin interaction. Both the membrane force and the stress fibre force will be described as acting in the negative *y*-direction orthogonal to the leading edge, i.e.

15 $$\begin{aligned} f_{0,x} = f_{L,x} = 0,\quad f_{0,y} = -f_{mem},\quad f_{L,y} = -f_{stress}. \end{aligned}$$

If these forces are modeled as constant, the Eq. (14) for the angle is decoupled from the remaining system. For an analysis of its dynamic behavior, we choose a model for the stiffness coefficients of the actin–myosin connection:

16 $$\begin{aligned} \mu ^{SM}(\varphi ) = \overline{\mu ^{SM}} \,(\varphi - \overline{\varphi })_+,\quad \mu ^{TM}(\varphi ) = \overline{\mu ^{TM}} \,(\varphi - \overline{\varphi })_+, \end{aligned}$$

with $\overline{\mu ^{SM}},\overline{\mu ^{TM}} >0$, $\varphi _0 < \overline{\varphi }< \pi$, and with the notation $(.)_+$ for the positive part.

Bistability can now be obtained with appropriate assumptions on the parameters. The right hand side of (14) can be written as

$$\begin{aligned}&\frac{24}{L^2} \cos \omega \left( \frac{f_{stress} - f_{mem}}{4} + h(\omega )\right) \quad \text{ with } \\&\quad h(\omega ) = \mu ^T(\pi -2\omega -\varphi _0) - 2\omega \overline{\mu ^{TM}} (\pi -2\omega -\overline{\varphi })_+. \end{aligned}$$

It is a simple exercise to prove:

### **Lemma 1**

If

17 $$\begin{aligned} \frac{\overline{\mu ^{TM}}}{\mu ^T}>\frac{\overline{\varphi }+\pi -2\varphi _0+2\sqrt{(\pi -\varphi _0)(\overline{\varphi }-\varphi _0)}}{(\pi -\overline{\varphi })^2}, \end{aligned}$$

then $h(\omega )$ as defined above has three simple zeroes $\omega _{10}$, $\omega _{20}$, $\omega _{30}$, satisfying

$$\begin{aligned} \frac{\pi }{2} > \omega _{10} = \frac{\pi -\varphi _0}{2} > \frac{\pi -\overline{\varphi }}{2} > \omega _{20} > \omega _{30} > 0. \end{aligned}$$

### **Theorem 2**

Under the assumptions of Lemma 1 and for $|f_{stress}-f_{mem}|$ small enough, the ordinary differential Eq. (14) with the forces given by (15) possesses four stationary solutions $\omega _j$, $j=0,\ldots ,3$ with

$$\begin{aligned} \omega _0=\pi /2 > \omega _1 = \frac{\pi -\varphi _0}{2} + \frac{f_{stress} - f_{mem}}{8\mu ^T} > \frac{\pi -\overline{\varphi }}{2} > \omega _2 > \omega _3 > 0, \end{aligned}$$

where $\omega _0$ and $\omega _2$ are unstable, and $\omega _1$ and $\omega _3$ are asymptotically stable.

Again the proof is straightforward. For the stable steady states, the lamellipodium has the constant protrusion speeds

$$\begin{aligned} \dot{y} = v \sin \omega _{1,3} - \frac{f_{stress} + f_{mem}}{\mu ^A L}. \end{aligned}$$

For the equilibrium angle $\omega _1$, we typically expect the speed to be positive. It is not affected by actin–myosin interaction. The smaller speed corresponding to $\omega _3$ might actually be negative due to membrane tension and stress fibres, i.e. the second stable state, where the lamellipodium is collapsed by actin–myosin interaction, might be retractive.

Finally, the steady states also produce lateral flow with constant speeds

$$\begin{aligned} \dot{x} = v\cos \omega _1 \quad \text{ and }\quad \dot{x} = \left( v - v^M \frac{4\mu ^{SM}\cos \omega _3}{\mu ^A + 4\mu ^S\cos \omega _3 + 4\mu ^{SM}\cos \omega _3}\right) \cos \omega _3, \end{aligned}$$

respectively, where in the collapsed state the lateral flow speed produced by polymerization is reduced by actin–myosin interaction.

## Coupling of two opposing lamellipodia–bistability

As a caricature of a cell fragment, we consider two back-to-back planar lamellipodia (see Fig. 1e). For notational convenience, the bottom lamellipodium is rotated by $180^\circ$ in the mathematical description. Therefore we consider two versions of the system (12)–(14) with unknowns $(x,y,\omega )$ and $(\hat{x}, \hat{y}, \hat{\omega })$. The assumption that the total forces exerted on the fragment by membrane tension and by stress fibres vanish, imply that (15) is used in both systems with the same values for $f_{mem}$ and $f_{stress}$. However, we allow the option that these forces are not constant but regulate the size of the fragment, measured by $y+\hat{y}$. We first consider the case of a constant given membrane force and a size dependent force by stress fibres:

$$\begin{aligned} \mathbf{Case}\,\,\mathbf{A:} \quad f_{mem} =\text{ const }, \quad f_{stress} = f_{stress}(y+\hat{y}). \end{aligned}$$

Typically $f_{stress}$ will be an increasing function, but the details are not important for our considerations.

Adding the Eq. (13) for *y* and $\hat{y}$ leads to a closed system of three equations for $y+\hat{y}$, $\omega$, and $\hat{\omega }$:

18 $$\begin{aligned} (\dot{y} + \dot{\hat{y}}) \mu ^A= & {} -\frac{2}{L}(f_{mem} + f_{stress}(y+\hat{y})) +\mu ^A v (\sin \omega + \sin \hat{\omega }) , \end{aligned}$$

19 $$\begin{aligned} \dot{\omega }\,g(\omega )= & {} \cos \omega \left( \frac{f_{stress}(y+\hat{y}) - f_{mem}}{4} + h(\omega )\right) , \end{aligned}$$

20 $$\begin{aligned} \dot{\hat{\omega }} \,g(\hat{\omega })= & {} \cos \hat{\omega } \left( \frac{f_{stress}(y+\hat{y}) - f_{mem}}{4} + h(\hat{\omega })\right) , \end{aligned}$$

with

$$\begin{aligned} g(\omega ) = \frac{L^2}{24} \left[ \mu ^A + 4\sin ^2\omega \cos \omega (\mu ^S + \mu ^{SM}(\pi -2\omega ))\right] . \end{aligned}$$

We shall prove that with appropriate assumptions on the data, the problem has 4 stable steady states.

### **Theorem 3**

Let the assumptions of Lemma 1 hold, let the function $f_{stress}$ be continuously differentiable with bounded positive derivative, and let $f_{mem}$, $\mu ^A vL$, and the Lipschitz constant of $f_{stress}$ be small enough. Then the system (18)–(20) has four stable steady states, satisfying

21 $$\begin{aligned}&\omega =\hat{\omega } = \omega _\text {CL}, \end{aligned}$$

22 $$\begin{aligned}&\omega =\hat{\omega } = \omega _\text {MY}, \end{aligned}$$

23 $$\begin{aligned}&\omega = \omega _\text {CL},\quad \hat{\omega } = \omega _\text {MY}, \end{aligned}$$

24 $$\begin{aligned}&\omega = \omega _\text {MY},\quad \hat{\omega } = \omega _\text {CL}, \end{aligned}$$

where the purely cross-link dominated state $\omega _\text {CL}$ and the myosin-influenced state $\omega _\text {MY}$ are given by:

$$\begin{aligned}&\omega _\text {CL}:=\omega _{10} + O(f_{mem} + \mu ^A vL)\,,\\&\omega _\text {MY}:=\omega _{30} + O(f_{mem} + \mu ^A vL)\,. \end{aligned}$$

### *Proof*

From (18) we obtain that steady states have to satisfy

25 $$\begin{aligned} f_{stress}(y+\hat{y}) = -f_{mem} + \frac{\mu ^A Lv}{2}(\sin \omega + \sin \hat{\omega }). \end{aligned}$$

This implies, again for stable steady states, $h(\omega ) = h(\hat{\omega }) = O(f_{mem} + \mu ^A vL)$. The existence of the four steady states is then a consequence of a straightforward perturbation argument. The coefficient matrix in the linearization of (18)–(20) can be written as

$$\begin{aligned} \left( \begin{array}{c@{\quad }c@{\quad }c} -2\kappa /(\mu ^A L)&{}v\cos \omega &{} v\cos \hat{\omega } \\ A\kappa &{} Ah^\prime (\omega )&{} 0 \\ \hat{A} \kappa &{} 0&{}\hat{A} h^\prime (\hat{\omega })\end{array}\right) , \end{aligned}$$

with positive constants *A* and $\hat{A}$, and with $0<\kappa = f_{stress}^\prime (y+\hat{y}) \ll 1$. A perturbation analysis of the eigenvalue problem for small $\kappa$ (i.e. formal expansion of eigenvalues in terms of powers of $\kappa$ and subsequent justification by a contraction argument) gives the eigenvalues

$$\begin{aligned} \lambda _1= & {} Ah^\prime (\omega ) + O(\kappa ),\quad \lambda _2 = \hat{A}h^\prime (\hat{\omega }) +O(\kappa ),\\ \lambda _3= & {} \kappa \left( -\frac{2}{\mu ^A L} + \frac{v\cos \omega }{h^\prime (\omega )} + \frac{v\cos \hat{\omega }}{h^\prime (\hat{\omega })}\right) + O(\kappa ^2), \end{aligned}$$

which are all negative at the four steady states for small enough $\kappa$, because of $h^\prime (\omega _{10}), h^\prime (\omega _{30}) < 0$. $\square$

For the steady states the protrusion speed of the fragment is constant and given by

26 $$\begin{aligned} \dot{y} = -\dot{\hat{y}} = \frac{v}{2}(\sin \omega - \sin \hat{\omega }). \end{aligned}$$

For the symmetric steady states (21), (22), the protrusion speeds vanish, hence they describe stationary cells (or fragments). The equilibrium angles in the lamellipodia in this case are either both affected by myosin, (22), or both result only from cross-link activity (21). The asymmetric steady states (23) and (24) describe a protruding, polarized cell. In both cases it consists of a collapsed cell rear, in which myosin is active ($\omega =\omega _\text {MY}$), and a cell front with a steeper equilibrium angle caused only by cross-link activity ($\omega =\omega _\text {CL}$).

Finally, we also mention the case of a constant stress fibre force and a size dependent membrane force:

$$\begin{aligned} \mathbf{Case}\,\,\mathbf{B:} \quad f_{stress} =\text{ const }, \quad f_{mem} = f_{mem}(y+\hat{y}). \end{aligned}$$

Without going through the details, we note that the qualitative results are the same and a theorem analogous to Theorem 3 can be proven.

## Parameter dependencies

The simplifications of the two preceding sections lead to several (testable) statements. This section can be seen as a discussion of the results so far.

*Protrusion speed versus adhesion strength* For the coupled lamellipodium of Sect. 6 the protrusion speed is given by (26). For a moving fragment, i.e. situation (23) or (24), we compute

$$\begin{aligned} \frac{d(\sin \omega _\text {CL} - \sin \omega _\text {MY})}{d\mu ^A} = \left( \cos \omega _\text {CL} - \frac{d\omega _\text {MY}}{d\omega _\text {CL}} \cos \omega _\text {MY}\right) \frac{d\omega _\text {CL}}{d\mu ^A}. \end{aligned}$$

Substitution of (25) into the stationary version of (19) (resp. (20)) shows that $d\omega _\text {CL}/d\mu ^A>0$. On the other hand, the difference of the stationary versions of (19) and (20) gives $d\omega _\text {MY}/d\omega _\text {CL} < 0$. Thus, the protrusion speed increases with adhesion strength. Since the results of Theorem 3 are restricted to relatively small adhesiveness, this is in agreement with experimental results (Barnhart et al. 2011) showing two regimes: for smaller adhesion strengths the cell speed is positively correlated with the adhesiveness of the ground. After reaching a maximum speed, the correlation is reversed for larger adhesion strengths.

**Fig. 2:**
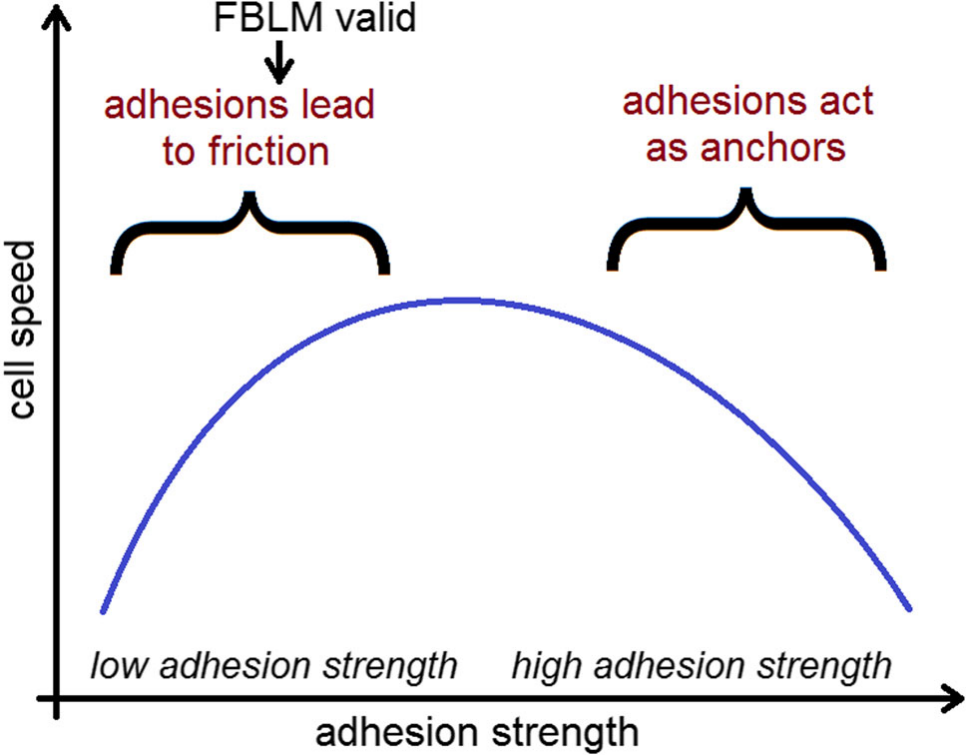
Influence on cell speed in different adhesive regimes

It is not clear if the FBLM in its present form is able to describe the second regime, which might be caused by long-lived focal adhesions, contradicting the assumption of rapid adhesion turnover. It should also be noted that it has been hypothesized (see Barnhart et al. 2011) that the reduction of cell speed on strongly adhesive ground is mainly due to biochemical reasons as opposed to purely mechanical effects. Figure 2 illustrates these considerations.

*Onset of myosin activity* The inequality (17) gives a criterion for the existence of a myosin influenced stable steady state. Myosin has to be strong enough compared to forces maintaining the branched actin network structure (e.g. Arp2 / 3, filamin, etc.). Only then is it able—either spontaneously or aided by a pushing force—to initiate the collapse of the network, which precedes bundle formation. Data like those presented in Reymann (2012) show that myosin activitiy is influenced by actin network structure. The model for myosin activity (16), used for the analyis in this work, can be interpreted as a limiting case, in which the activity is set to zero for unfavorable angles. From the approximation

$$\begin{aligned} \overline{\mu ^{TM}} (\pi - \overline{\varphi })^2 > 4\mu ^T (\pi - \varphi _0) \end{aligned}$$

of (17) for $\overline{\varphi }$ close to $\pi$, it is easily seen that if the interval for the myosin angle is small, this needs to be compensated by a large stiffness of the myosin filament in order to initiate symmetry breaking. In our model myosin is necessary for the onset of movement, i.e. polarization of originally symmetric fragments. In Yam (2007) the number of stationary cells spontaneously initiating motility within a given time interval was reduced from 45 to 10 % by inhibiting myosin using blebbistatin, which supports our findings.

*Protrusion speed versus myosin strength* In the presented model two parameters characterize myosin strength, the elasticity coefficients of myosin filament stretching $\overline{\mu ^{SM}}$ and bending $\overline{ \mu ^{TM}}$. Whereas $\overline{\mu ^{SM}}$ has no influence on the equilibrium angle and therefore also not on the protrusion speed, larger values of $\overline{\mu ^{TM}}$ lead to faster cells (easily seen in the expression for $h(\omega )$). This is consistent with experimental results found in Barnhart et al. (2011) where myosin contraction was either inhibited using blebbistatin (leading to slower cells at least for low to medium adhesion strengths) or enhanced using calyculin A (leading to faster cells in all adhesive regimes).

**Table 1:** Parameter values

Var.	Meaning	Value	Comment
*L*	Filament length	$8\,\mathrm{\upmu m}$	Order of magnitude as in Verkhovsky et al. (1999)
$A_0$	Equilibrium inner area	$300\,\mathrm{\upmu m}^2$	Order of magnitude as in Verkhovsky et al. (1999)
*N*	Total filament number	9000	Order of magnitude as in Koestler et al. (2008)
$\mu ^B$	Bending elasticity	$0.7\,\mathrm{pN\,\upmu m}^2$	10 times higher than in Gittes et al. (1993)
$\mu ^A$	Macroscopic friction caused by adhesions	$0.14\, \text {pN min} \,\mathrm{\upmu m}^{-2}$	Measurements in Li et al. (2003), Oberhauser et al. (2002), estimation and calculations in Ölz et al. (2008), Ölz and Schmeiser (2010a, b)
*v*	Polymerization speed	$3\,\mathrm{\upmu m}\,\text {min}^{-1}$	In biological range
$\varphi _0$	Equilibrium cross-link angle	$70^\circ$	Equal to the branching angle
$\mu ^S$	Cross-link stretching constant	$4.2\times 10^{-3}\, \mathrm{pN\, min}\, \,\mathrm{\upmu m}^{-1}$	
$\mu ^T$	Cross-link twisting constant	$4.2\times 10^{-3}\, \mathrm{pN\,\,\upmu m}$	
$v^M$	Myosin velocity	$1\,\mathrm{\upmu m}\,\text {min}^{-1}$	Order of magnitudes as in Svitkina et al. (1997)
$\overline{\varphi }$	Myosin cut-off	$100^{\circ }$	
$\overline{\mu ^{SM}}$	Myosin stretching constant	$4.2\times 10^{-3}\, \text {pN min}\, \,\mathrm{\upmu m}^{-1}$	
$\overline{\mu ^{TM}}$	Myosin twisting constant	$1.4 \times 10^{-2}\,\mathrm{pN\,\upmu m}$	Motivated by Lemma 1, Simulation 1
		$1.8 \times 10^{-2}\,\mathrm{pN}\,\mathrm{\upmu m}$	Motivated by Lemma 1, Simulation 2
$\mu ^\text {stress}$	Stress fiber force	$5\times 10^{-2} \,\mathrm{pN \upmu m}^{-1}$	

## Simulations with the full model

In this section we demonstrate that with the additional term describing myosin within the lamellipodium, the model is able to produce cells/cell fragments that, depending on the initial conditions, will either remain stationary or start moving. In contrast to the simulations presented in Manhart et al. (2015a, b), here the movement is achieved without a continuing external signal and without varying the polymerization speed. In the simulation, we work with the full model (6)–(8) and not with the simplifications introduced in Sects. 3 and 4. However, the qualitative results of Sect. 6 will be reproduced.

*Parameter values* Parameter values are chosen as in Manhart et al. (2015a) with the following exceptions and additions: we work with a constant filament density $\eta =1$ in parameter space, which means that the filament number remains constant with branching and capping always in equilibrium. No pointed ends appear within the simulation region, which corresponds to a fixed filament length of $L=8\,\mathrm{\upmu m}$. The polymerization speed is fixed at the constant value $v=3\,\mathrm{\upmu m}\, \text {min}^{-1}$. In Svitkina et al. (1997) it has been observed that myosin speckles that are formed in the lamellipodium drift inwards with time. This indicates that the myosin velocity has to be smaller than the polymerization speed. We therefore chose $v^M=1\,\mathrm{\upmu m}\, \text {min}^{-1}$. Motivated by Reymann (2012) we assume that myosin can only act on actin filaments if the angle between the filaments is larger than $\overline{\varphi }=100^\circ$. For the stiffness parameters of stretching and twisting the cross-links and myosin good estimates are hard to obtain, since their exact concentration in the lamellipodium is difficult to determine. Motivated by Lemma 1 we chose the myosin twisting force larger than the cross-link twisting force (see Table 1), but of the same order of magnitude. Additionally we increased the bending stiffness by a factor 10 in order to get closer to the analytical case examined in Sect. 3. Here we concentrated on the case of size control via stress fiber forces from the inside, i.e. the membrane force was set to zero. The stress fiber force was chosen to be $f_{stress}=\mu ^{IP}(A-A_0)_+$, where *A* is the current size of the area surrounded by the lamellipodium, $A_0$ is the equilibrium inner area and $\mu ^{IP}$ is the corresponding (inner pulling) force constant. In order not to distort the direction of the filaments, we let the stress fibers pull only tangentially on the pointed ends. The numerical parameters are chosen as in Manhart et al. (2015a), i.e. 36 discretization nodes in $\alpha$ direction and 9 nodes in *s*-direction. The timestep used is $2\times 10^{-3}$ min.

**Fig. 3:**
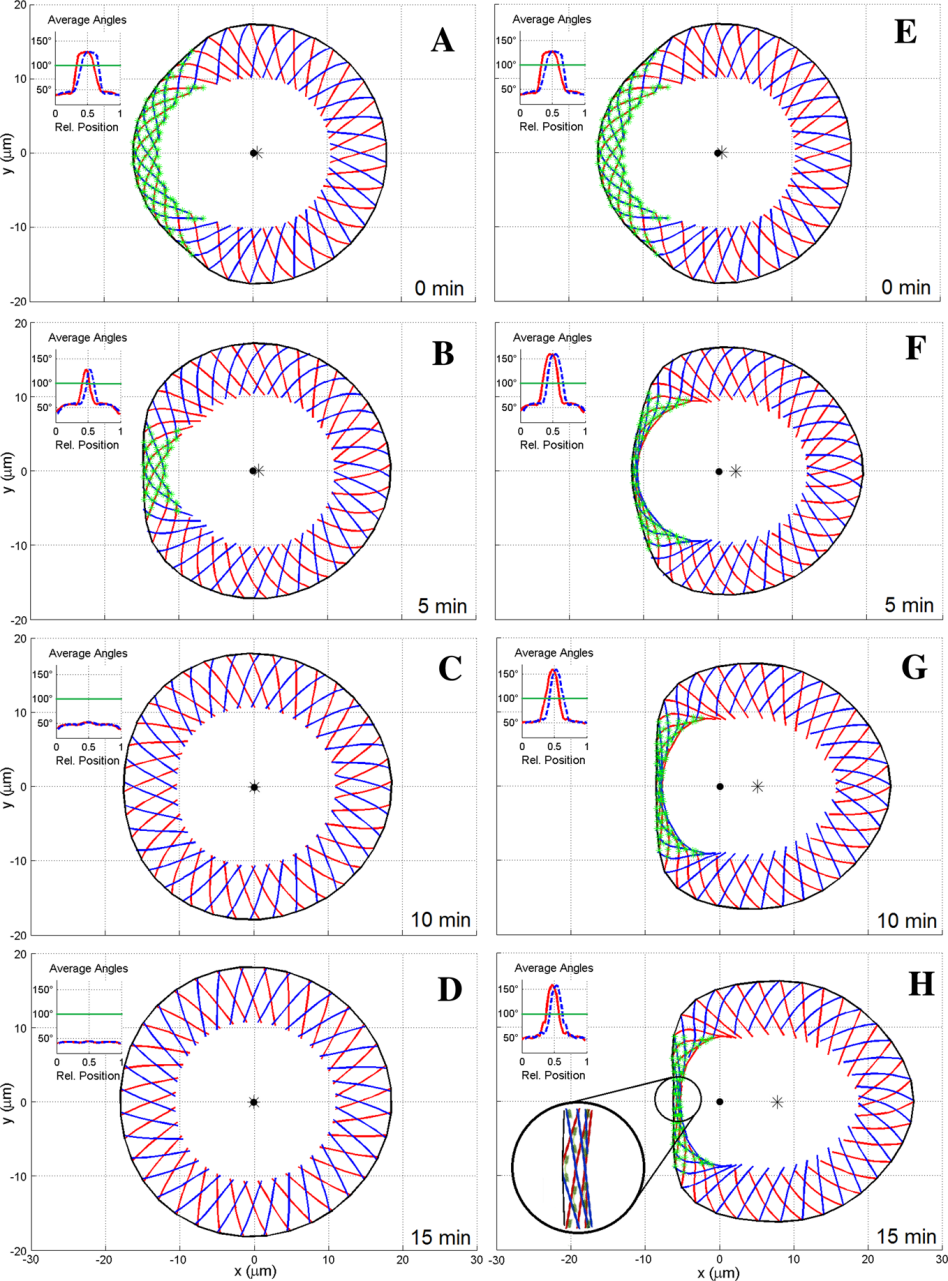
Cell view with clockwise filaments in *blue* and anti-clockwise filaments in *red*. *Green stars* in the lamellipodium mark the area where myosin is active. The *black dot marks* the origin, the *black star* the cell’s center of mass. The *insets* show the angles between the filaments of the different families, averaged along each filament, parametrized along the membrane with 0 being at the very right and going counterclockwise. *Blue* (*dashed*) *lines* refer to the clockwise filament family, *red* (*solid*) *lines* to the anti-clockwise filament family. The *horizontal green line* at $100^\circ$ marks the myosin cut-off value $\overline{\varphi }$. **a–d** Time series with $\mu ^{TM}=1.4 \times 10^{-2}\,\mathrm{pN}\,\mathrm{\upmu m}$. Myosin is initially active in the *left part* of the cell, but is not strong enough to stay there. Eventually the cell goes back to the stationary cross-link dominated equilibrium. **e–h** Time series with $\mu ^{TM}=1.8 \times 10^{-2}\,\mathrm{pN}\,\mathrm{\upmu m}$. Myosin is initially active in the *left part* of the cell, where a myosin/cross-link equilibrium emerges. Since the right of the cell is unaffected by this, the cell moves to the *right*. Parameters as in Table 1 (color figure online)

*Simulation results* Figure 3 shows the evolution of the cell in two different numerical experiments over a timespan of 15 min. In both cases identical initial conditions were used, in which the left side of the cell is deformed. This could be caused either by internal fluctuations or by a pushing force from the left, two situations known to lead to symmetry breaking in fragments (Verkhovsky et al. 1999). Both simulations use the same set of parameters, with the exception of the value of the myosin twisting constant, $\mu ^{TM}$, which is smaller in the left column than in the right column. In Fig. 3a–d it can be observed that even though the filaments on the left initially lie anti-parallel enough for myosin to act on them, myosin does not establish itself there permanently. Lemma 1 gives a possible explanation for this: If myosin is too weak compared to the cross-links, there is no myosin/cross-link-equilibrium, hence the cell reverts back to a purely cross-link dominated state. This situation corresponds to the steady state (21) in Theorem 3.

The case of a larger myosin twisting constant is depicted in Fig. 3e–h. In situation case a bundle-precursor is formed on the left, whereas the right stays myosin free. This allows the cell to change to a moving steady state (see the Supplementary Material for a movie) with a collapsed lamellipodium at the back and a non-collapsed lamellipodium at the front, a situation described by the steady states (23) and (24) in Theorem 3. One can also observe the contraction of the rear bundle leading to a more half-moon shaped cell. Clearly if the initial deformations are so small that no myosin can attach, the cell always reverts back to the stationary state. This refers to a situation where the fluctuations or pushing force are too small to cause symmetry breaking.

## Discussion and outlook

In this work, we extended the FBLM introduced in Manhart et al. (2015a), Ölz and Schmeiser (2010a) by a description of actin–myosin interaction. The limit of large bending stiffness led to a simplified model for rigid filaments. The additional simplification of a planar lamellipodium reduces the model to a small ODE system for the center of mass of a reference filament and the angle with the membrane. A caricature of a cell fragment has been described by considering two versions of the model coupled by membrane and stress fiber forces. Bistability has been shown in the asymptotic regime of relatively small coupling forces and adhesion strength. Since these results are almost explicit, various parameter dependencies could be obtained. Furthermore, we carried out numerical simulations based on the full FBLM supporting the main analytical result of bistable behaviour.

Our model is able to qualitatively reproduce the observed bistability of cells and cell fragments (Verkhovsky et al. 1999). Since myosin has been shown to be effective only if the angle between the filaments is large enough (Reymann 2012), our specific modeling of the myosin effect to be angle-dependent seems to be a reasonable choice.

In the numerical simulations we have shown that for an initially slightly asymmetric cell, one of two stable steady states is attained, depending on the parameters for myosin and cross-links. If myosin is too weak to exert considerable twisting forces, the effects from cross-links on the local angles between the filaments dominate and the cell reverts back to a symmetric nonmoving shape. On the other hand, if myosin forces are strong enough, a bundle precursor forms at the ’rear’ (the location of initial asymmetry), and the cell starts moving. What is apparent in these simulations is that myosin tends to locate at the back of the lamellipodium (away from the membrane). This is also in good agreement with experimental findings. It is remarkable that both for the analytical setting as well as in the numerical simulations, movement is an self-organized behavior, once an initial asymmetry has been established. It is known that keratocytes exhibit little if any chemotaxis, i.e. directed movement to outward cues.

An important issue remains to be addressed: The analytical model, dealing only with pieces of lamellipodium at the front and at the rear of the cell, avoids the transition zone separating the two parts of the lamellipodium with an intact network on the one hand and, on the other hand, compression to a rear bundle. It can be expected that due to lateral flow, actin filaments are drawn into the bundle in these transition regions. However, we expect that this happens with the pointed ends first entering the bundle, as opposed to the simulations presented here. A model supporting the necessary change of orientation in the transition zone is not available so far, and its derivation is the subject of ongoing work.

## Electronic supplementary material

Below is the link to the electronic supplementary material.

Supplementary material 1 (avi 2632 KB)
